# A case of Amyand hernia at the Central Hospital of Yaounde and review of the literature

**DOI:** 10.1186/s40792-023-01632-9

**Published:** 2023-05-16

**Authors:** J. C. Fouda, Philip Fernandez Owon’Abessolo, Bob Dorcas Nyanit, Junior Barthelemy Mekeme Mekeme, P. Savom, A. Ranibel, A. A. Mbassi, G. Bwelle, G. A. Bang, P. J. Fouda, Faustin Mouafo Tambo, A. Essomba

**Affiliations:** 1 Yaounde Central Hospital, Yaounde, Cameroon; 2 Department of Surgery and Specialties, Faculty of Medicine and Biomedical Sciences, University of Yaounde I, Yaounde, Cameroon; 3 University of Douala, Douala, Cameroon

## Abstract

**Introduction:**

Amyand's hernia is defined as an inguinal hernia, containing the appendix in the hernia sac. It is a rare form of hernia. Its management is increasingly codified.

**Clinical history:**

A 5-year-old patient with a non-remarkable past history was brought for consultation with an intermittent inguino-scrotal swelling and discomfort. Clinical examination revealed a non-tender inguino-scrotal swelling with positive transillumination. A conclusion of a communicating hydrocele was made; hence, an indication for surgery. Per operatively, we had as findings the appendix present within, and linked to the hernia sac. We performed an appendectomy and a high ligation of the hernia sac. The post-operative evolution was favourable. Anatomopathological analysis revealed a catarrhal appendix.

**Conclusion:**

Amyand's hernia remains a rare pathology that can be seen in children with a persistent peritoneo-vaginal canal. Dissection of the hernia sac must be carried out carefully since it is most often discovered intraoperatively and accidental injury to the appendix, which is attached to the wall of the hernia sac can lead to serious complications.

## Introduction

Amyand's hernia is defined as an inguinal hernia, containing the appendix within the hernia sac [1]. The incidence of this rare disease is up to 1% (0.19–1.7%) of all cases of inguinal hernias [1]. Inflammation of the appendix in the inguinal sac is rarer, accounting for 0.1% (0.07–0.13%) of all cases of Amyand's hernia [1] and 2% of appendectomies in the neonatal and childhood population [2].

## Case report

A 5-year-old AD child presented with intermittent swelling of his right scrotum. There was no specific history and the systemic review was unremarkable. The physical examination showed good general state and satisfactory vital signs. Examination of the scrotum revealed a right scrotal swelling which was unfolded, non-inflammatory with a positive transillumination test and present silk glove sign. The right testis was difficult to palpate and the left testis was normal. The rest of the examination was unremarkable.

Summarily, a persistence of the right peritoneal canal was retained as conclusion with the anatomical entity being a communicating hydrocele in a 5-year-old subject. An operability test was performed which was normal. A preanesthetic consultation was requested (ASA 1 and Althemeier 1).

**Fig. 1:**
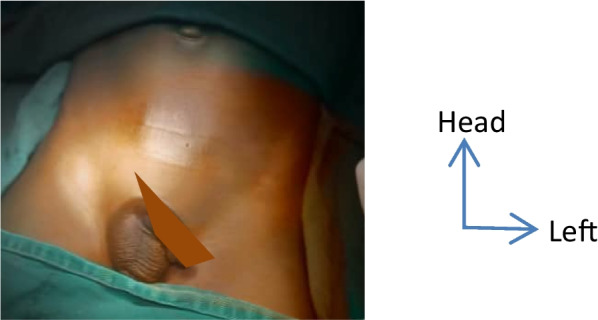
Installation

Surgically, the patient was installed on dorsal decubitus under general anaesthesia and oro-tracheal intubation (Fig. 1).

An incision was made on the lower abdominal fold, the superficial fascia pulled off and a fasciotomy performed.

The cord was protected and dissection of the cremasteric fascia was done. This was followed by isolation of the sac from the elements of the spermatic cord; essentially, the vas deferens.

**Fig. 2:**
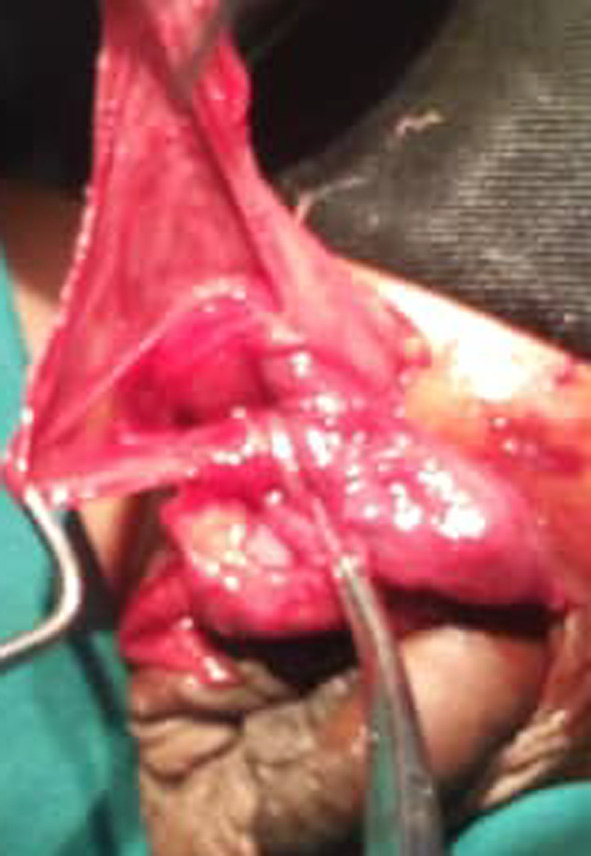
The peritoneo-vaginal canal

**Fig. 3:**
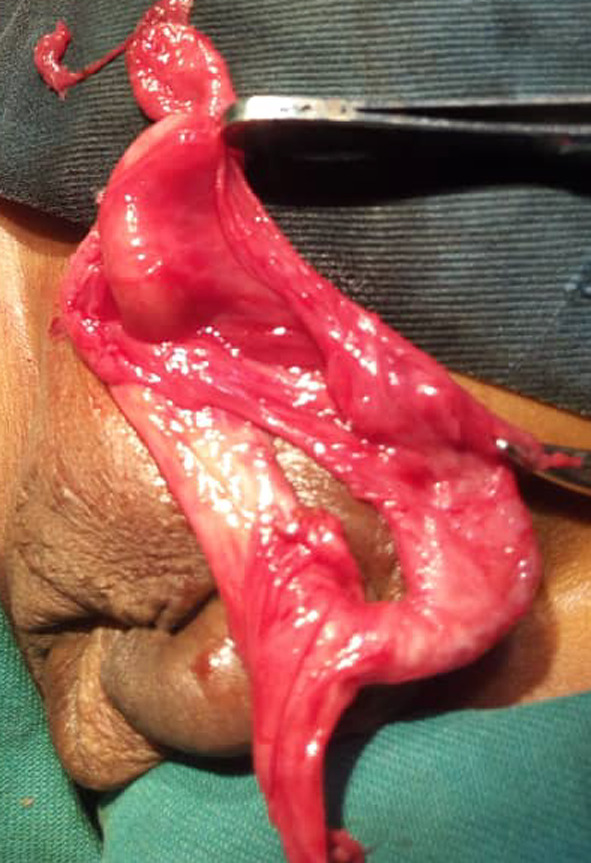
Appendix visualised in the the peritoneo-vaginal canal

**Fig. 4:**
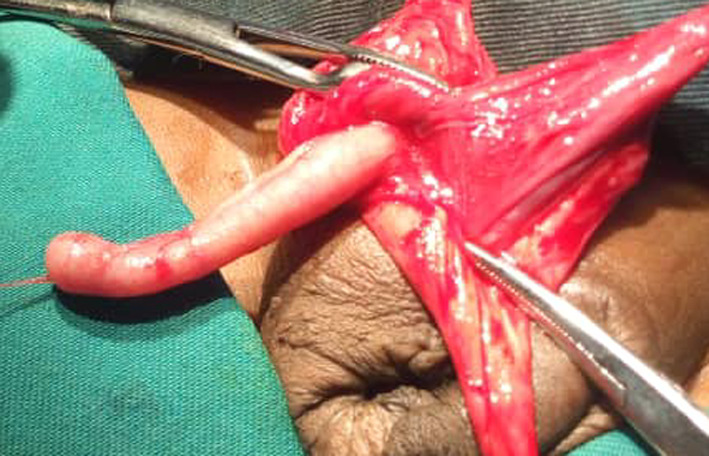
Insulated appendix of the bag

**Fig. 5:**
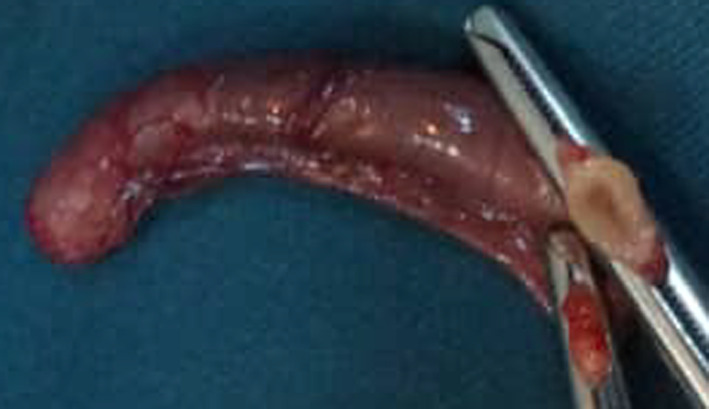
Appendectomy specimen

Dissection of the sac permitted us to discover an erectile vermiform appendix adhered to the wall of the hernia sac (Figs. 2, 3, 4). We performed an appendectomy after careful dissection of the wall of the hernia sac in order to isolate the appendix (Fig. 5) and carry out a high ligation of the hernia sac, followed by a layer-by-layer closure and a dry dressing.

Anatomopathological analysis showed a peritoneal hernia sac and a catarrhal appendix. The post-operative evolution was favourable. The patient was discharged 5 days after surgery. He was reviewed 3 months later without any remarkable finding.

## Discussion

Amyand's hernia is a rare type of inguinal hernia where the appendix is located within and/or incarcerated in the hernia sac [3]; in our case, the appendix was adhered to the wall of the hernia sac. It is more common in boys due to the higher incidence of right inguinal hernias [4], which is the case in our patient.

Amyand's hernia constitutes 1% of all inguinal hernias hence rare. In 0.3% of cases the appendix is inflamed [1, 5] as in our case and the presence of acute appendicitis represents 0.1% of all appendicitis [6, 7]. In our patient, the appendix was catarrhal [8]. Regarding pathophysiology, the appendix becomes inflamed inside the hernia, and this may be secondary to the vascular involvement caused by the pressure of the hernial neck, triggering the inflammatory process and the subsequent bacterial proliferation, without ruling out luminal obstruction by fecaliths, ganglionic hypertrophy, parasites, or other causes [4] however due to the presence of the inguinal ring, the inflammatory process of the appendix in AH may not extend into the abdominal cavity and may be limited to the inguinal canal, affecting the cecum or the base of the ring if they are also within the hernia sac [9].

Amyand's hernia has been reported in patients aged 03 weeks to 92 years, with a threefold greater likelihood of being diagnosed in children than in adults. Regarding pathophysiology, the appendix migrates into the hernia sac, where some authors state that a fibrous connection between the appendix (retrocecal) and the testicles added to the persistence of processes vaginalis favour the passage of the appendix into the inguinal canal and this would be the reason for the higher incidence of this condition in children and premature infants [4]. Indeed, our patient had a persistent peritoneo-vaginal canal (scrotal swelling with positive transillumination in the patient). It is generally accepted that as a result of the elevation of abdominal pressure due to the abdominal muscle contraction, the appendix enters into the hernia sac, and in the advanced stage bacterial overgrowth and inflammation develop in the appendix by disruption of blood supply [5]. Unlike adults, the fact that the ileocaecal region is not fixed in children can be considered a key point in treatment and diagnosis.

The hernia was in the right inguinal region. This presentation is in line with literature, which finds most cases of Amyand's hernia on the right side [10]. Most of the cases occur on the right side, probably as a consequence of the normal anatomical position of the appendix and also because right-sided inguinal hernias are more common than left-sided hernias [11]. Although Amyand's hernia has also been reported on the left side, this is rare and may be associated with situs inversus, intestinal malrotation or a mobile cecum [12, 13].

Amyand's hernia is more frequent in inguinal hernias [14], which is the case in our patient. Inguinal hernias are found above the inguinal ligament and superior to the pubic tubercle. They may be direct or indirect. Direct inguinal hernias lie anteromedial and inferior to the lower epigastric vessels; whereas indirect hernias project posterolaterally and superior to the vessels [15]. In children, it will be an indirect hernia because of the persistence of the peritoneo-vaginal canal compared to adults.

The difficulty in diagnosis is due to the variation in symptoms that the patient presents depending on the state of the appendix (normal, incarcerated or perforated) [8]. In this patient, the appendix was inflamed and the patient had discomfort in addition to scrotal swelling with positive transillumination. Abdominal exam, physical signs, lab results and imaging are not always helpful in making differential diagnosis [4]. Imaging is not recommended by most surgeons unless the inguinal hernia is not easily reducible or incarcerated [1].

Therefore, we can conclude on type two based on the Amyand's hernia classification after modification of Rikki and Losanoff and Basson in Amyand's hernia.

In most cases, as in our case, the diagnosis is made intraoperatively [2, 16]. The appendix was adhered to the hernia sac. Pathophysiologically, Abu-Dalu and Urca suggested that the appendix becomes more vulnerable to trauma in Amyand's hernia causing microtrauma and inflammation, eventually retained by adhesions and hence, the appendix may adhere to the hernial sac [5, 6]. The presence of the appendix in the hernia sac predisposes to the development of adhesions between the serous membrane and the hernia sac [5, 16].

Appendectomy at the same time as hernia repair is still a debate [17].

Losanoff and Basson proposed a classification for the establishment of a therapeutic framework (Table 1) which was modified by Rikki (Table 2) [18].

**Table 1:** Losanoff and Basson classification of Amyand's hernia

Classification	Description	Management
Type 1	Normal appendix in an inguinal hernia	Hernia reduction and mesh replacement
Type 2	Acute appendicitis in inguinal hernia without abdominal sepsis	Appendectomy, primary hernia repair without prosthesis
Type 3	Acute appendicitis in an inguinal hernia with sepsis of the abdominal and/or peritoneal wall	Laparotomy and appendectomy, primary hernia repair without prosthesis
Type 4	Acute appendicitis in inguinal hernia with concomitant abdominal disease	Same as type 3 plus concomitant disease management

**Table 2:** Amyand hernia classification after Rikki modification

Classification	Description	Management
Type 1	Normal appendix in an inguinal hernia	Hernia reduction and mesh replacement
Type 2	Acute appendicitis in inguinal hernia without abdominal sepsis	Appendectomy, primary hernia repair without prosthesis
Type 3	Acute appendicitis in an inguinal hernia with sepsis of the abdominal and/or peritoneal wall	Laparotomy and appendectomy, primary hernia repair without prosthesis
Type 4	Acute appendicitis in inguinal hernia with concomitant abdominal disease	Same as type 3 plus concomitant disease management
Type 5a	Normal appendix in an incisional hernia	Hernia reduction, primary hernia repair including mesh replacement
Type 5b	Acute appendicitis in incisional hernia without peritonitis	Hernia appendectomy, primary closure of fascial space, no prosthetic hernia repair
Type 5c	Acute appendicitis within an incisional hernia with peritonitis or sepsis of the abdominal wall or in relation to a previous surgical procedure	Same as type 4

**Fig. 6:**
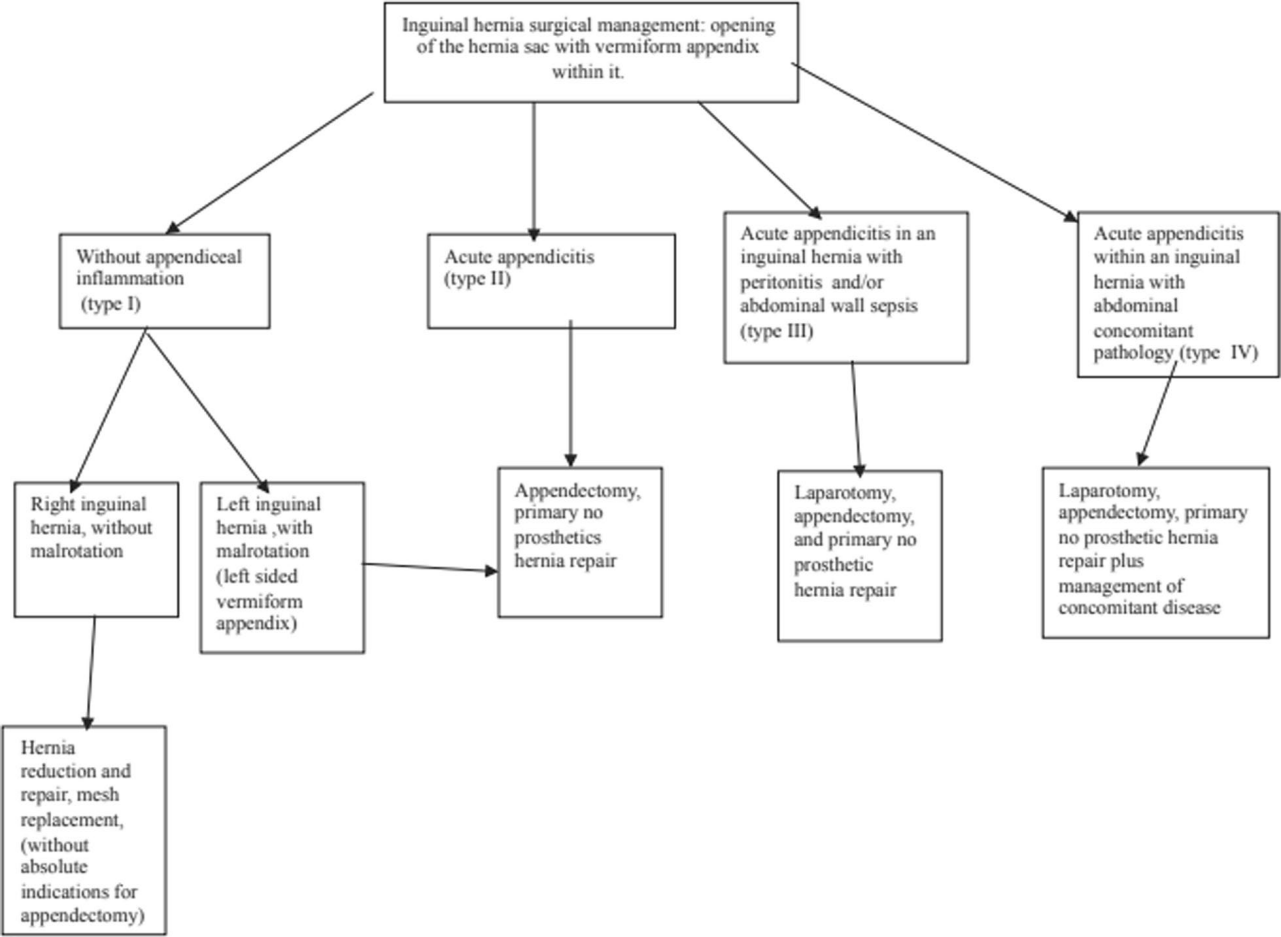
Therapeutic Algorithmic of Amyand’s hernia

Based on this classification, we can classify our patient as type 2. And our management consists of the following diagram (Fig. 6) [1].

The classic hernia repair is performed by pushing back and ligating the sac. In adult patients, additional repair with mesh is recommended. However, it has been reported that especially in cases with a perforated appendix, repair using mesh may increase the risk of infection and adversely affect wound healing, with the potential danger of recurrence [9].

The decision to preserve a normal appendix during AH repair is currently personalised. The decisions are partly based on the surgeon's competence to perform appendicectomy without complications, future occurrence of appendiceal disease and the usefulness of a preserved appendix for future conduit surgeries [19].

In adult, there is the debate on the best way to strengthen the posterior wall contrary to the child [19].

Throughout the literature, the management of Amyand's hernia in both children and adults follows the classification of Losanoff and Basson.

Our patient had a post-operative favourable evolution but literature review reports a mortality rate of 15 to 30% due to severe sepsis [4–6].

Due to the rarity of Amyand’s hernia and the wide variety of its presentation, each case study and review article sheds light into new and useful information regarding its treatment and diagnosis [4].

## Conclusion

Due to its rare nature, Amyand’s hernia diagnosis remains a per operative finding, hence a demand for more diligents by surgeons. The choice of surgery (appendicectomy and herniotomy or only herniotomy) is surgeon dependent, based on the presentation and difficulties encountered as well as the therapeutic options put in place.

## Data Availability

Not applicable for that section.
